# The effect of hemoperfusion on the outcome, clinical and laboratory findings of patients with severe COVID-19: a retrospective study

**DOI:** 10.1016/j.nmni.2021.100937

**Published:** 2021-09-02

**Authors:** A. Soleimani, S.M. Moeini Taba, S. Hasibi Taheri, A.H. Loghman, M. Shayestehpour

**Affiliations:** 1) Department of Internal Medicine, Faculty of MedicineKashan University of Medical Sciences, Kashan, Iran; 2) Autoimmune Diseases Research CenterKashan University of Medical Sciences, Kashan, Iran; 3) Department of Microbiology and Immunology, Faculty of Medicine, Kashan University of Medical Sciences, Kashan, Iran

**Keywords:** COVID-19, hemoperfusion, outcome, SARS-CoV-2, treatment

## Abstract

Hemoperfusion is a method of blood filtering to remove toxins and inflammatory factors. Cytokine storms and high levels of inflammatory factors play a role in the pathogenesis of coronavirus disease 2019 (COVID-19). The present study aims to evaluate the effect of hemoperfusion on the clinical and laboratory findings and outcomes of patients with severe COVID-19. Forty-eight patients with severe COVID-19 and a positive PCR test, who were admitted to the intensive care unit (ICU), participated in the study. All patients were treated by routine treatment protocol for COVID-19. Hemoperfusion was performed for 24 patients in addition to treatment with conventional antiviral therapies. The other 24 patients made up the control group. Demographic data, laboratory findings and patient outcomes before and after treatment were retrospectively collected and analysed. There was no significnt difference in mortality or length of hospital stays between the control group and the hemoperfusion group. The breathing rate (P-vaalue = 0.001) and heart beat rate (P-value = 0.028) of patients decreased after hemoperfusion. The hemoperfusion resulted in a significant increase in the SpO2 levels and a significant decrease in the CRP of patients compared to the conventional treatment (P-value = 0.009). Hemoperfusion can improve respiratory distress. It can reduce the CRP in patients with severe COVID-19 but has no effect on mortality.

## Introduction

Coronavirus disease 2019 (COVID-19) is an infectious disease caused by the severe acute respiratory syndrome coronavirus 2 (SARS-CoV-2). This acute viral infection is often associated with respiratory failure, pneumonia, acute respiratory distress syndrome (ARDS) and sepsis [1]. According to the latest statistics reported by the World Health Organization (WHO), more than 70 million people have been infected with SARS-CoV-2 worldwide. The new coronavirus is responsible for more than 1.6 million deaths. To date, SARS-CoV-2 has infected nearly 1.4 million people in Iran and killed more than 53,000 [2].

Although most patients with COVID-19 have a mild form of the disease (81%), it is accompanied by severe and life-threatening manifestations with a mortality rate of 49%. Studies have shown that the levels of various cytokines such as IL-2R, IL-6, IL-10, IL-2, IL-7, GCSF and TNF-α are significantly higher in patients with severe COVID-19 compared with patients who have a mild disease [3,4]. Mortality is also higher in patients with higher IL-6 levels than in others. This evidence suggests the role of cytokine release syndrome (CRS) in the severity of disease and the outcome of patients [5].

Hemoperfusion is a method of filtering blood. The target molecules are removed from the blood when it passes through a column that contains adsorbent particles, which are either activated charcoal or resin. In this method, toxins or some inflammatory mediators are removed from the blood using special cartridges. Hemoperfusion can decrease IL-6 and IL-8 levels in patients with sepsis and can decrease the mortality rate [6,7].

Inflammatory cytokines in patients with severe COVID-19 are high; therefore, hemoperfusion may improve the patients’ condition. Several studies have evaluated the therapeutic effect of hemoperfusion on COVID-19 patients and have obtained varying findings [[8], [9], [10]]. Further studies must be performed to confirm the useful effect of this method for treating patients with severe COVID-19. The present study aimed to investigate the effect of hemoperfusion on the outcomes and clinical and laboratory findings for patients with severe COVID-19.

## Methods

This retrospective study was carried out on patients with severe COVID-19 admitted to Shahid Beheshti Hospital in Kashan between February 2020 to March 2020. The Ethics Committee of Kashan University of Medical Sciences approved the study (IR.KAUMS.MEDNT.REC.1399.117). Patients admitted to ICU with severe COVID-19, including a positive PCR test result and a positive chest CT scan, participated in the study. All patients were treated by routine treatment protocol for COVID19. The patients were divided into two groups. The first group underwent hemoperfusion in the inflammatory phase of the disease in addition to the current treatment, while the second group only received conventional treatment. The patients underwent hemoperfusion three times with HA330 and HA280 filters for four hours. Inclusion criteria were: (1) having at least two clinical criteria, including respiratory rate more than 30 times per minute, oxygen saturation (SPO2) less than 85%, or intermittent fever.(2) having at least three laboratory criteria, including PaO2<60 mmHg, PaO2/FiO2<200, CRP>60 mg/dL, ferritin> 2000, an elevated D-dimer, fibrinogen <150 mg/dL and bicytopenia (platelet counts below 100,000, hemoglobin below 9 g/dL and a lymphocyte count below 1100/μl). All patients with a platelet count below 20,000 were excluded from the study.

Laboratory tests were performed one day before and 72 hours after hemoperfusion. Demographic data, laboratory findings and outcomes of patients before and after treatment were retrospectively collected. To evaluate the effectiveness of hemoperfusion, PaO2, and vital signs before and after hemoperfusion (only in hemoperfusion group), as well as SpO2, ESR, CRP, fibrinogen, ferritin and lymphocytes of patients in both groups were extracted and compared.

### Statistical analysis

Data analysed by SPSS version 22. Demographic characteristics and outcomes of patients between two groups were compared by Chi-square test, student’s t-test and Mann–Whitney *U* test. Paired t-test and Wilcoxon signed-rank test were also used to evaluate the findings before and after the intervention in the hemoperfusion group. Normal distribution was checked using the Shapiro–Wilk test. A p-value of less than 0.05 was considered significant.

## Results

Forty-eight patients with severe COVID-19 participated in this study. Twenty-four patients were placed in the hemoperfusion group, and the same number of patients were placed in the control group. The control group ranged in age from 33 to 97 years. The hemoperfusion group had an age range of 27 to 85 years. Demographic characteristics and outcomes of patients are summarized in Table 1. There was no significant difference in mortality (P-value = 0.330) and length of hospital stays (P-value = 0.883) between the control and hemoperfusion groups.

**Table 1:** Demographic characteristics and outcome of the patients

Variable	Control (n = 24)	Hemoperfusion (n = 24)	P
Age, years	62.17 ± 17.28	52.58 ± 14.60	**0.044**^a^
Gender			
Male	10 (41.7%)	12 (50%)	0.562^c^
Female	14 (58.3%)	12 (50%)	
Outcome			
Death	8 (33.3%)	5 (20.8%)	0.330^c^
Discharge	16 (66.7%)	19 (79.2%)	
Length of stay, days	17.83 ± 8.99	19.21 ± 11.66	0.883^b^

Data are presented as mean ± SD and n (%).

A p-value less than 0.05 is statistically significant.

a Independent t-test.

b Mann-Whitney *U* test.

c Pearson chi-square.

Comparison of vital signs and PaO2 before and after hemoperfusion (Table 2) showed that the mean number of breaths after hemoperfusion decreased from 39.50 to 23.94 (P-value = 0.001). Heart rate decreased from 92.04 to 85.46 (P-value = 0.028). In addition, PaO2 increased from 54.58 to 83.80 (P-value <0.001).

**Table 2:** Vital signs and PaO_2_ before and after hemoperfusion

Variable	Before	After	P
PaO_2_, mmHg	54.58 ± 17.42	83.80 ± 29.82	**<0.001**^b^
SBP, mmHg	114.58 ± 14.66	114.54 ± 13.52	0.924^b^
DBP, mmHg	72.08 ± 8.33	69.79 ± 10.27	0.367^b^
RR, breath/min	39.50 ± 5.57	23.94 ± 9.46	**0.001**^b^
HR, pulse/min	92.04 ± 17.61	85.46 ± 15.98	**0.028**^a^
Temperature, °C oral	36.82 ± 0.53	36.55 ± 0.51	0.055^b^

Abbreviations: PaO_2_, partial pressure of oxygen in arterial blood; SBP, systolic blood pressure; DBP, diastolic blood pressure; RR, respiratory rate; HR, heart rate.

Data are presented as mean ± SD.

A p-value less than 0.05 is statistically significant.

a Paired samples t-test.

b Wilcoxon signed-rank test.

Clinical and laboratory findings before and after treatment of patients with severe COVID-19 are summarized in Table 3. Analysis of the results showed that hemoperfusion had resulted in a more significant increase in the SpO2 of patients compared to those that received only conventional treatment (P-value = 0.009). In addition, the CRP is more significantly decreased through performing hemoperfusion in comparison with the use of conventional treatment.

**Table 3:** Clinical and laboratory findings before and after treatment

Variables	Control (n = 24)			P^a^	Hemoperfusion (n = 24)			P^a^	P^b^
	Before	After	Difference		Before	After	Difference		
SpO_2_, %	89.75 ± 5.58	92.94 ± 2.70	3.19 ± 5.76	**0.043**^c^	80.73 ± 12.74	91.68 ± 7.12	10.95 ± 11.22	**0.001**^d^	**0.009**^e^
CRP, mg/dL	85.38 ± 56.74	45.29 ± 47.41	–40.08 ± 62.10	**0.004**^d^	160.96 ± 80.12	67.08 ± 54.77	–93.88 ± 91.32	**< 0.001**^d^	**0.021**^e^
ESR, mm/hour	52.00 ± 32.31	56.96 ± 29.13	4.96 ± 32.65	0.658^d^	65.13 ± 32.62	55.75 ± 32.09	–9.38 ± 38.00	0.239^c^	0.168^e^
Fibrinogen, mg/dL	1337.63 ± 365.22	1080.67 ± 378.75	–256.96 ± 567.93	**0.026**^d^	648.21 ± 472.00	446.17 ± 327.02	–202.04 ± 425.61	0.052^d^	0.564^f^
Ferritin, ng/mL	2472.71 ± 183.89	1413.08 ± 760.66	–1059.63 ± 726.73	**< 0.001**^d^	1957.46 ± 551.38	570.29 ± 457.49	–1387.17 ± 641.57	**< 0.001**^d^	0.081^f^
Lymphocytes, per mm^3^	997.54 ± 427.96	1136.33 ± 675.38	138.79 ± 602.50	0.484^d^	734.21 ± 301.37	1165.66 ± 649.33	431.45 ± 745.72	**0.009**^c^	0.142^e^

Abbreviations: SpO_2_, oxygen saturation; CRP, C-reactive protein; ESR, erythrocyte sedimentation rate.

Data are presented as mean ± SD.

A p-value less than 0.05 is statistically significant.

a P value for comparing before and after values.

b P value for comparing difference between control and hemoperfusion group.

c Paired samples t-test.

d Wilcoxon signed-rank test.

e Independent t-test.

f Mann-Whitney *U* test.

## Discussion

Results of the present study showed a significant increase in the level of SpO2 and a significant decrease in the CRP level among the hemoperfusion group compared to the control group. In addition, a significant decrease in respiration rate, heart rate and an increase in the level of PaO2 was observed after hemoperfusion.

Several studies have evaluated the therapeutic effect of hemoperfusion on COVID-19 patients. In the case of COVID-19 reported by Dastan et al., the patient underwent hemoperfusion three times with an HA380 cartridge. Subsequently, the level of SpO2 increased, and the patient’s clinical condition improved. The levels of IL-1, IL-6, IL-8 and TNF-α decreased [11]. The findings of this study confirm the results of our study on the improvement of vital signs and the recovery of patients with a severe type of COVID-19 after hemoperfusion. Asgharpour et al. reported an improvement in 60% of patients with COVID-19 after hemoperfusion [8]. Similarly to the present study, they observed a significant decrease in the CRP level and an increase in the SpO2 level of patients. In a study conducted on patients with sepsis and acute lung injury, hemoperfusion reduced IL-1, IL-6 and IL-8 levels [12]. They reported that hemoperfusion had not decreased the mortality rate. Hemoperfusion therapy in our study also had no effect on the mortality rate of patients with severe COVID-19. Katagiri et al. performed hemoperfusion for two days on twelve patients with COVID-19 who had a PaO2/FiO2 level less than 300. Disease severity was reduced in 58.3% of patients, and the PaO2/FiO2 level was increased [13].

Although it is expected that hemoperfusion decreases inflammatory cytokines, it could not reduce the mortality rate and level of IL-6 in patients with septic shock, as shown in the study by Schädler et al. [14]. In the study of Bernardi et al., hemoperfusion could only significantly decrease IL-10. They did not observe a significant change in the levels of IL-1β, IL-6, IL-18, or TNF-α, or in mortality rate after hemoperfusion [15]. Shadvar et al. treated 8 patients with severe COVID-19 by hemoperfusion. The CRP, D-Dimer and ferritin levels in patients decreased. In our study, hemoperfusion significantly decreased the CRP but not the ferritin level.

Several studies have shown that coronaviruses can induce an excessive and prolonged cytokine/chemokine response (cytokine storm), which results in acute lung injury (ALI), acute respiratory distress syndrome (ARDS), high morbidity and mortality due to immunopathology. Previous studies have found an association between an increase in levels of IL-2, IL-6, IL-7, IL-10 and TNF-α and the severity or mortality of COVID-19. This evidence suggests the role of cytokine release syndrome (CRS) in disease severity and the recovery of patients. Therefore, it seems that the CRS can play an essential role in the pathogenesis of COVID-19. Hence, hemoperfusion can benefit patients with COVID-19 by removing these cytokines from the body and inhibiting CRS. It prevents lung tissue damage by preventing apoptosis of lung cells and increases the SpO2 level. Following patients’ improvement and inflammation reduction, ferritin and CRP levels are also reduced.

There are several drugs that inhibit some of the cytokines, such as Tocilizumab (IL-6 receptor inhibitor) and anakinra (IL-1β inhibitor). However, the use of other alternative methods such as hemoperfusion is useful when patients are in critical condition. Anti-cytokine drugs act specifically on one cytokine, but hemoperfusion can decrease different cytokines simultaneously.

The present study had several limitations. In this retrospective study, the level of inflammatory cytokines such as IL-6 was not measured at the time of intervention. Other factors influencing mortality, such as comorbidities, have not been considered.

## Conclusion

Hemoperfusion could improve respiratory distress and reduced the CRP in patients with severe COVID-19 but has no effect on mortality. It is recommended that further studies are performed with larger sample sizes.

## Credit author statement

Alireza Soleimani: Conceptualization, Methodology. Sara Hasibi-Taheri: Data curation, Writing- Original draft preparation. Amir Hossein Loghman: Visualization, Investigation. Seyed-Masoud Moeini Taba: Supervision. Mohammad Shayestehpour: Writing- Reviewing and Editing.

## Transparency declaration

The authors declare that there is no conflict of interest.

This study was funded and supported by Kashan University of Medical Sciences (grant: 99126).

## Statement of ethics

This study was approved by the Ethics Committee of Kashan University of Medical Sciences (IR.KAUMS.MEDNT.REC.1399.117).
